# Targeted agents in patients with progressive glioblastoma—A systematic meta‐analysis of randomized clinical trials

**DOI:** 10.1002/cam4.7362

**Published:** 2024-06-21

**Authors:** Franziska Maria Ippen, Angelika Scherm, Tobias Kessler, Peter Hau, Sarina Agkatsev, Hansjörg Baurecht, Wolfgang Wick, Helge Knüttel, Michael F. Leitzmann, Corinna Seliger‐Behme

**Affiliations:** ^1^ Department of Neurology and Neurooncology Program, National Center for Tumor Diseases University Hospital Heidelberg Heidelberg Germany; ^2^ Wilhelm Sander‐NeuroOncology Unit and Department of Neurology University Hospital Regensburg Regensburg Germany; ^3^ Clinical Cooperation Unit Neurooncology, German Cancer Consortium (DKTK) German Cancer Research Center (DKFZ) Heidelberg Germany; ^4^ Department of Neurology University Hospital Knappschaftskrankenhaus Bochum, Ruhr University Bochum Bochum Germany; ^5^ Institute of Epidemiology and Preventive Medicine, University Hospital Regensburg Regensburg Germany; ^6^ University Library, University of Regensburg Regensburg Germany

**Keywords:** glioblastoma, meta‐analysis, progressive, targeted therapies

## Abstract

**Background:**

Glioblastoma (GB) is the most common malignant primary brain tumor in adults and is associated with a poor prognosis. Current treatment guidelines outline the standard of care for patients with newly diagnosed GB; however, there is currently no well‐established consensus for the treatment of progressive GB. With this systematic meta‐analysis of recently published randomized controlled trials (RCTs), we aim to establish evidence on targeted agents in the treatment of patients with progressive GB.

**Material and Methods:**

We conducted searches across the Cochrane Library, Pubmed, MEDLINE (Ovid), ClinicalTrials.gov, WHO‘s International Clinical Trials Registry Platform and Google Scholar, encompassing the time span from 1954 to 2022, aiming to identify RCTs evaluating targeted therapies in patients with progressive GB. In order to perform a random‐effects meta‐analysis, we extracted hazard ratios (HRs) of overall survival (OS) and progression‐free survival (PFS).

**Results:**

We included 16 RCTs (*n* = 3025 patients) in the systematic meta‐analysis. Formally, regorafenib (RR 0.50; 95% CI 0.33–0.75), Depatux‐M + TMZ (RR 0.66; 95% CI 0.47–0.93) and rindopepimut + bevacizumab (RR 0.53; 95% CI 0.32–0.88) were associated with an improved OS compared to the control arm. The combination of bevacizumab + CCNU (RR = 0.49; 95% CI 0.35–0.69) and regorafenib (RR 0.65; 95% CI 0.44–0.95) were formally associated with improved PFS.

**Conclusions:**

The aim of this systematic meta‐analysis was to establish evidence for the use of targeted therapies in progressive GB. While some studies demonstrated benefits for OS and/or PFS, those results have to be interpreted with caution as most studies had major methodological weaknesses, including potential differences in sample size, trial design, or the initial distribution of prognostic factors.

## 1 INTRODUCTION

Glioblastoma (GB) is the most common primary brain tumor of the central nervous system in adult cancer patients, accounting for up to 60%–70% of all diagnosed malignant gliomas with an annual incidence of 3.23 cases per 100,000 population in the United States.^1^ Affected patients are generally facing a poor prognosis, with a median survival of 3–4 months^2^ if the tumor remains untreated and up to a median overall survival (mOS) of 15–26 months^3^, ^4^ if patients are receiving multimodality treatment. The current standard of care at initial diagnosis consists of surgical resection or biopsy followed by radio‐chemotherapy with temozolomide (referred to as the Stupp protocol)^5^ with or without the addition of tumor‐treating fields.^6^ Disease recurrence occurs in almost all GB patients and is associated with a very limited mOS of 9 months and a 12‐months overall survival (OS) of 14% of affected patients.^7^, ^8^ However, treatment strategies for patients with progressive GB are less well established and treatment options are mainly based on prior therapy, age, Karnofsky Performance Status (KPS), O^6^‐methylguanin‐DNA‐methyltransferase (MGMT) promoter methylation status and patterns of disease progression.^9^ The three mainly pursued strategies of medical treatment for progressive GB after first‐line treatment with radiochemotherapy with temozolomide include nitrosourea‐based regimens such as lomustine as monotherapy or in combination, alternative dosing regimens of temozolomide, and the use of bevacizumab, especially in case of additional radiation necrosis or treatment‐resistant edema.^9^, ^10^ The profound heterogeneity of GB, not only just between different patients, but also within a single tumor^11^ has been posing extraordinary challenges in regards to sufficient treatment, but has also provided the basis for targeting different signaling pathways involved in tumor progression with the perspective of a potential benefit of more personalized treatment options.

In this systematic review and consecutive meta‐analysis of randomized clinical trials on targeted therapies in patients with progressive GB, we aimed to provide an overview of the current status and highest level of evidence on the role of targeted therapies in this particular patient cohort. Furthermore, we intended to identify subgroups of patients with progressive GB who might benefit more from targeted treatment options with regards to overall and progression‐free survival (PFS).

## 2 MATERIALS AND METHODS

### 2.1 Study design and systematic literature search

This meta‐analysis was performed in accordance with the Preferred Reporting Items for Systematic Reviews and Meta‐Analyses (PRISMA)^12^, ^13^ and the MOOSE guidelines^14^ (see PRISMA checklistData S1).

MEDLINE (Ovid)/Pubmed, Cochrane Library, ClinicalTrials.gov, WHO's International Clinical Trials Registry Platform and Google Scholar were searched for articles on targeted therapies in patients with GBs from the date of inception or availability up to 07/27/2021 (WHO ICTRP: 03/18/2022). The concept GB was used in conjunction with the Boolean operator AND with search filters for randomized clinical trials. For each of these concepts, we chose relevant subject headings and text words to allow for maximal search sensitivity.

To begin with, a primary search strategy was developed for MEDLINE. For other databases the subject headings and syntax were adapted. We strived to abide by the PRESS guideline,^15^ but a peer review of the search strategy was not performed. A draft search strategy for MEDLINE was published elsewhere.^16^ Additionally, we searched for further studies among the reference lists of included articles.

In order to de‐duplicate and process the records from the database searches, these were imported into the EndNote reference management software. This method has been published previously by Bramer et al.^17^ At first two researchers (F.M.I., A.S.) independently screened the titles. Subsequently, the previously extracted abstracts were checked for their relevance. To ascertain their eligibility, full‐text versions of the records that met the predefined inclusion criteria were obtained. The same was done for records that had to be checked for relevance to the topic. This eligibility screening was also performed independently by two researchers (F.M.I., A.S.). In the event of a disagreement, the ultimate decision was made by a third reviewer (C.S.). In the case of an exclusion of an article, the reasons were documented in detail.

### 2.2 Inclusion and exclusion criteria

To be considered for inclusion in the meta‐analysis, studies had to have analyzed patients with progressive GB who had been treated with targeted therapies compared to one of either treatment strategies in the control group (if available): (1) (alternative dosing regimens of) temozolomide (TMZ), (2) nitrosourea‐based treatment (for example lomustine [CCNU], carmustine [BCNU], fotemustine), (3) bevacizumab, (4) combinations of the treatment strategies mentioned before and with other established treatment options (for example procarbazine, lomustine and vincristine [PCV]), or different dosing schemes of the experimental treatment arm (for example, pembrolizumab or axitinib) or (5) placebo, if available after the initial search strategy. We only included prospective randomized controlled trials (RCTs) (either phase II or phase III), that analyzed the following statistical outcome parameters: mOS, median progression‐free survival (mPFS), PFS at 6 months (PFS‐6), HR for death or HR for progression, and 95% Confidence Intervals (CIs). Studies that reported on patients under the age of 18, nonhuman research, and articles in a language other than English were excluded. Targeted treatments included medications directed against growth factors and their receptors (EGF(R), VEGF(R) (KDR and FLT1), FGF(R), PDGF(R), HGF(R)/c‐MET, IGF‐1(R), TGF‐ß, c‐kit), signaling pathways (Ras/(B)Raf/MEK/MAPK(ERK), PI3K/Akt/mTOR, PKCß), cell cycle regulators/DNA repair mechanisms (MDM2, TP53, CDK4/6, RB1, PARP, HDA1c), checkpoint inhibitors (PD‐1, PDL‐1, CTLA‐4), and others (RET, IDH, Myc). Small molecule kinase inhibitors, antibodies and vaccines were considered to be targeted drugs. The following therapies and treatments, however, were excluded from further analysis: intratumoral or topic therapies (e.g., Gliadel wafers), oncolytic viral/antiviral/retroviral treatments (e.g., TOCA 511/FC, Ganciclovir, etc.), solely blood–brain‐barrier (BBB) permeability increasing drugs (e.g., RMP‐7) or repurposed drugs not directly targeting cancer‐associated pathways (e.g., losartan).

### 2.3 Data extraction

Two of the authors independently extracted data on trial design (phase and randomization), substances, treatment regimen, target, number of patients, study geographic region, length of follow‐up, mOS, OS at 1 year (OS‐12), mPFS, PFS at 6 months (PFS‐6) and 1 year (PFS‐12), HR for death, HR for progression, CIs, and histology or molecular subtype. In case of availability of the additional investigations of patient subgroups, these data were also extracted and evaluated.^16^

### 2.4 Statistical analysis

HRs for death and tumor progression were interpreted as relative risk estimates (RRs). Subsequently, the natural logarithm of those risk estimates log(RR_i_) was calculated with the corresponding standard error s_i_ = d_i_/1.96, with di representing the maximum of [log(upper 95% CI bound of RR_i_)‐log(RR_i_)] and [log(RR_i_)‐log(lower 95% CI bound of RR_i_)].

Potential publication bias was evaluated with funnel plots, Begg's rank correlation test,^18^ and Egger's regression test.^19^ In order to assess the heterogeneity among risk estimates the Q‐statistic and the *I* ^2^‐statistic^20^ were applied. A random‐effects meta‐analysis was performed.^21^ Pooled RRs with 95% CIs of targeted agents were calculated as compared to the most widely used treatment strategies mentioned above among patients with progressive GB. The meta‐analysis was calculated using the metafor, robumeta and dplyr packages in R 3.6.1 (The R Foundation for Statistical Computing, Vienna, Austria). Statistical tests were two‐sided and statistical significance was based on the 5% significance level.

## 3 RESULTS

### 3.1 Search results obtained from databases and register

**FIGURE 1:**
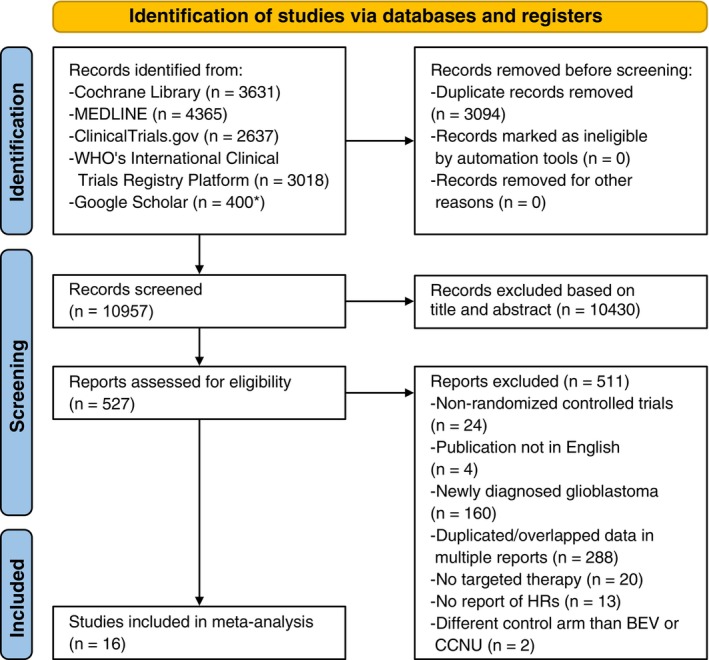
PRISMA flow diagram illustrating the literature selection process of this systematic meta‐analysis. (*) Google Scholar: 400 records were downloaded for each one of the searches in 2019 and 2021. BEV, Bevacizumab; CCNU, Lomustine.

The steps of our literature perusal are displayed in the PRISMA flow chart (see Figure 1). We received a total of 14,051 results for evaluation ranging from the year 1954 to 2022. After deduplication 10,957 were further analyzed. However, after examining the title and abstract, a total of 10,430 references were excluded. Therefore, 527 articles were left for full‐text evaluation. As a result of not meeting the inclusion criteria, 511 of the aforementioned 527 articles were excluded. Thus, 16 studies were included in our meta‐analysis of studies published between 2010 and 2020. The search strategies based on a linear search algorithm have already been published.^16^

### 3.2 Characteristics of included studies

This meta‐analysis included 3025 patients with progressive GB. Of these, 1824 patients were assessed as part of the experimental arm versus 1201 patients as part of the control arm. Among the studies included, we identified seven studies comparing the experimental treatment arm to treatment with CCNU in the control arm. Of these seven trials, two analyzed VEGF‐inhibition (bevacizumab) in combination with CCNU,^22^, ^23^ one evaluated combined VEGFR‐, PDGFR‐, and FGFR‐inhibition (cediranib) with or without the addition of CCNU,^24^ one considered multi‐tyrosine kinase inhibition with regorafenib,^25^ one analyzed protein kinase C inhibition (enzastaurin),^26^ one TGF‐β‐inhibition (galunisertib)^27^ with or without the addition of CCNU and one EGFR‐inhibition (Depatux‐M) with and without the addition of TMZ.^28^

Nine studies were identified comparing the experimental treatment arm to bevacizumab treatment in the control arm. Among those studies, two analyzed VEGF‐inhibition (bevacizumab) in the experimental arm either in combination with either carboplatin^29^ or CCNU,^30^ one trial assessed EGFRvIII‐directed immunotherapy (rindopepimut)^31^ in combination with bevacizumab, one study evaluated combined MET‐ and VEGF‐inhibition (onartuzumab)^32^ in combination with bevacizumab, one considered multi‐tyrosine kinase inhibition with dasatinib^33^ in combination with bevacizumab, one evaluated the viral‐based anticancer gene therapy ofranergene obadenovec (VB‐111)^34^ in combination with bevacizumab, angiopoietin (Ang)‐TIE2 system inhibition (trebananib)^35^ in combination with bevacizumab was assessed in one trial, treatment with the PD‐1 directed antibody nivolumab alone versus treatment with bevacizumab was analyzed in the Checkmate‐143 trial^36^ and the histone deacetylase inhibitor (HDAC) vorinostat in combination was assessed compared to bevacizumab alone.^37^

While three studies^24^, ^27^, ^28^ consisted of trials with two experimental arms, in order to exclude bias and avoid overestimating the respective control arm, we focused on one arm reported in those studies. Table 1 summarizes the basic characteristics of the studies that were included in this meta‐analysis.

**TABLE 1:** Baseline characteristics of the included studies.

Substance	Author	Year	Study name	Phase	Targets	Intervention		Number		mOS (months)		OS‐12 (%)		mPFS (months)		PFS‐6 (%)	
						Ex	Co	Ex	Co	Ex	Co	Ex	Co	Ex	Co	Ex	Co
Experimental treatment versus CCNU																	
Bevacizumab	Brandes	2018	TAMIGA	II	VEGF	Bevacizumab/CCNU	CCNU	61	62	6,4	5,5	11,7	16,5	2,3	1,8	9	3
Bevacizumab	Wick	2017		III	VEGF	Bevacizumab/CCNU	CCNU	288	149	9,1	8,6	31,5	34,1	4,2	1,5	28	17
Cediranib	Batchelor	2013	REGAL	III	VEGFR, PDGFR, FGFR	Cediranib	CCNU	131	65	8	9,8	30	41	3	2,7	16	25
Cediranib	Batchelor	2013	REGAL	III	VEGFR, PDGFR, FGFR	Cediranib/CCNU	CCNU	129	65	9,4	9,8	38	41	4,1	2,7	35	25
Regorafenib	Lombardi	2019	REGOMA	II	VEGFR, TIE2, KIT, RET, RAF‐1, BRAF, BRAFV600E, PDGFR, FGFR	Regorafenib	CCNU	59	60	7,4	5,6	38,9	15	2	1,9	16,9	8,3
Enzastaurin	Wick	2010		III	Proteinkinase C	Enzastaurin	CCNU	174	92	6,6	7,1	15	25	1,5	1,6	11,1	19
Galunisertib	Brandes	2016		II	TGF‐β	Galunisertib	CCNU	39	40	8	7,5	27	33	1,8	1,9	15	6
Galunisertib	Brandes	2016		II	TGF‐β	Galunisertib/CCNU	CCNU	79	40	6,7	7,5	29	33	1,8	1,9	6	6
Depatux‐M	Van den Bent	2019	INTELLANCE2	II	EGFR	Depatux‐M/TMZ	CCNU	88	86 (61LOM, 25 TMZ)	9,6	8,2	39,7	28,2	2,7	1,9	25	15
Depatux‐M	Van den Bent	2020	INTELLANCE2	II	EGFR	Depatux‐M	CCNU	86	86 (61LOM, 25 TMZ)	7,9	8,2	26,7	28,2	1,9	1,9	8	15
Experimental treatment versus Bevacizumab																	
Bevacizumab	Field	2015	CABARET	II	VEGF	Bevacizumab/Carboplatin	BEV	60	62	6,9	7,5	15	25	3,5	3,5	15	18
Bevacizumab	Weathers	2016		II	VEGF	Bevacizumab/CCNU	BEV	33	36	9,6	8,3	52	35	4,3	4,1	36,4	23,6
Rindopepimut	Reardon	2020	REACT	II	EGFRvIII	Rindopepimut/Bevacizumab	BEV	36	37	11,6	9,3	45	31	3,7	3,7	28	16
Onartuzumab	Cloughesy	2017	GO27819	II	MET, VEGF	Onartuzumab/Bevacizumab	BEV	64	65	8,8	12,6	22	50	3,9	2,9	33,9	29
Dasatinib	Galanis	2019		II	PDGFR, SRC, c‐KIT, BCR‐ABL	Dasatinib/Bevacizumab	BEV	83	38	7,3	7,7	22	27	3,2	3,2	28,9	18,4
VB‐111 (Ofranergene obadenovec)	Cloughesy	2019	GLOBE	III	VEGF	VB‐111/Bevacizumab	BEV	128	128	6,8	7,9	25,3	24,9	3,4	3,7	22	30
Trebananib	Lee	2020		II	VEGF, Ang1/2	Trebananib/Bevacizumab	BEV	57	58	7,5	11,5	52	31	4,2	4,8	22,6	41,1
Nivolumab	Reardon	2020	Checkmate‐143	III	PD‐1	Nivolumab	BEV	182	185	9,8	10	41,8	42	1,5	3,5	15,7	29,6
Vorinostat	Puduvalli	2020		II	HDAC, VEGF	Vorinostat/Bevacizumab	BEV	47	38	7,8	9,3	29	28	3,7	3,9	25	28

Abbreviations: Ang1/2, Angiopoietins 1 and 2; BCR‐ABL, Philadelphia chromosome (BCR‐ABL fusion gene), formed by fusion of the 3′ sequences from ABL1 (Abelson) gene at 9q34 to the 5′ portion of the BCR (breakpoint cluster region) gene sequences at 22q11; BEV, Bevacizumab; BRAF, v‐Raf murine sarcoma viral oncogene homolog B; BRAFV600E = mutation of the BRAF gene in which valine (V) is substituted by glutamic acid (E) at amino acid 600; Co, control; CCNU, Lomustine; EGFR, Epidermal growth factor receptor; EGFRvIII, Epidermal growth factor receptor variant three; Ex, experimental; FGFR, Fibroblast growth factor receptor; HDAC, Histone‐Deacetylase; c‐KIT/ KIT, KIT proto‐oncogene, receptor tyrosine kinase; MET, mesenchymal–epithelial transition receptor tyrosine kinase; mOS, median overall survival; mPFS, median progression‐free survival; PD‐1, Programmed cell death protein 1; PDGFR, Platelet‐derived growth factor receptor; RAF‐1, Raf‐1 proto‐oncogene, serine/threonine kinase; RET, Rearranged during transfection receptor tyrosine kinase; SRC, Src nonreceptor tyrosine kinase; TGF‐β, Transforming growth factor beta; TIE2, Angiopoietin‐1 receptor; TMZ, Temozolomide; VEGF, Vascular endothelial growth factor.

### 3.3 Outcome parameters—Overall survival

For the evaluation of OS 15 studies were considered, which was assessed in three separate subgroups: experimental treatment versus CCNU; experimental treatment + CCNU/TMZ versus CCNU and experimental treatment versus bevacizumab. Of the initial 16 studies in our meta‐analysis, one trial by Weathers et al.^30^ did not mention HRs for OS. Of note, just one study tested nivolumab against bevacizumab alone,^36^ all other studies that chose a control arm with bevacizumab combined the experimental treatment with bevacizumab itself.

**FIGURE 2:**
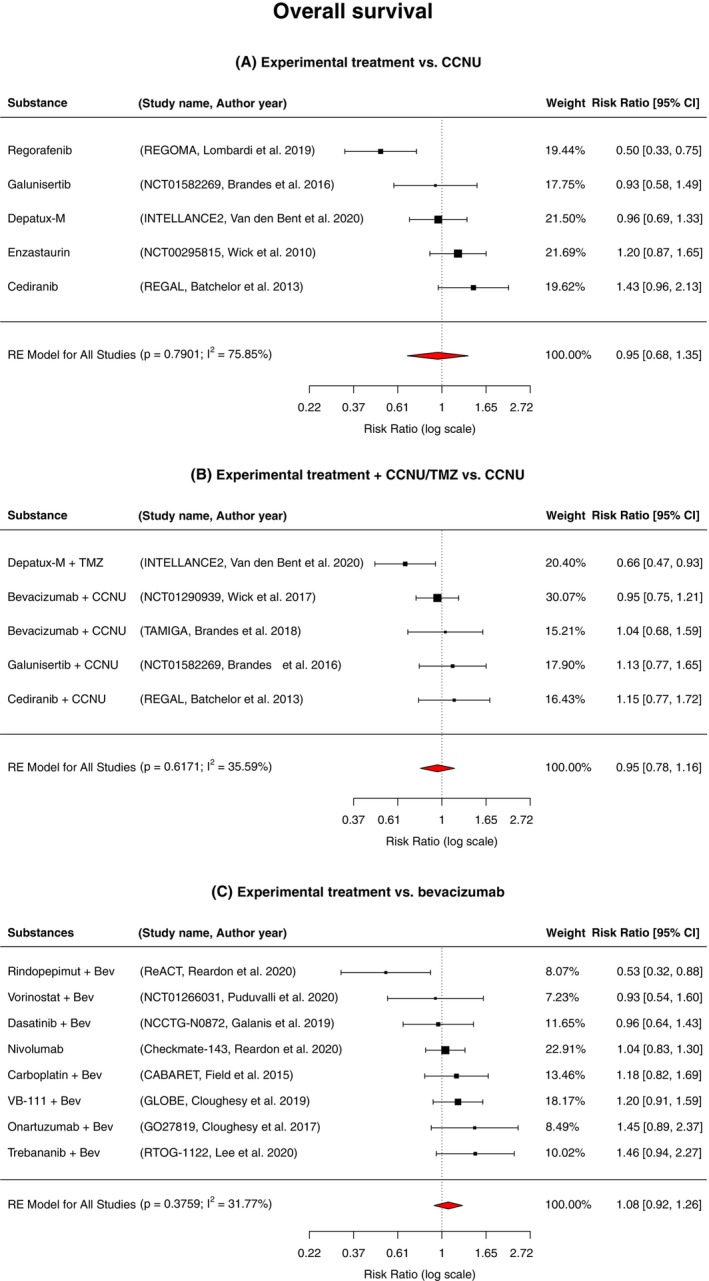
Forest plot displaying the pooled estimated risk ratio (red diamond) for overall survival across 15 RCTs of treatment of progressive GBs with experimental treatment versus (A) CCNU monotherapy, (B) experimental treatment + CCNU/TMZ versus CCNU monotherapy and (C) experimental treatment versus bevacizumab; Bev, Bevacizumab; CCNU, Lomustine; RE, risk estimate; TMZ, Temozolomide; VB‐111, Ofranergene obadenovec.

The random‐effect meta‐analysis showed a nonsignificantly reduced mortality risk of 0.95 (95% CI 0.68–1.35, *p* = 0.7901) for patients with progressive GB treated with targeted therapy compared to CCNU (*n* = 832; Figure 2A). A similar total mortality risk of 0.95 (95% CI 0.78–1.16, *p* = 0.6171) was observed for patients who received treatment with a targeted therapy + CCNU compared to CCNU alone (*n* = 1047, Figure 2B). Furthermore, a mortality risk of 1.08 (95% CI 0.92–1.26, *p* = 0.3759) demonstrated that there was no significant difference of mortality for patients who were treated with a targeted therapy compared to bevacizumab alone (*n* = 1268, Figure 2C). A considerable to moderate heterogeneity between studies was observed (*I* ^2^ = 75.85%; 35.59% and 31.77%, respectively), while only substantial heterogeneity revealed statistical significance (*p* = 0.0035; 0.1864 and 0.0925, respectively).

Next, separate analyses for each molecular target were conducted in the three different subgroups (Figures [Link], [Link], [Link]). The molecular targets consisted of VEGF(R), EGFR/EGFRvIII, MET, PDGFR, chemotherapy/viral therapy, Ang1/2 (Angiopoietins 1 and 2), PD‐1, HDAC, TGF‐β and protein kinase C. In the subgroup with targeted agents versus CCNU the only study resulting in a significant survival benefit in this subgroup was the REGOMA‐trial assessing treatment with the multikinase‐inhibitor regorafenib^25^ (RR 0.50; 95% CI 0.33–0.75; *p* = 0.0009, *n* = 119), while treatment with the multikinase‐inhibitor cediranib even showed an increased risk of death (RR 1.43; 95% CI 0.96–2.13; *p* = 0.1000, *n* = 196). In the experimental treatment + CCNU/TMZ versus CCNU subgroup, the only survival benefit was found for EGFR‐inhibition with Depatux‐ M+ TMZ in the INTELLANCE2‐trial^28^ (RR 0.66; 95% CI 0.47–0.93; *p* = 0.0176, *n* = 174). With regards to the experimental treatment versus bevacizumab subgroup, only EGFRvIII‐inhibition with rindopepimut + bevacizumab demonstrated a survival benefit (RR 0.53; 95% CI 0.32–0.88; *p* = 0.0141, *n* = 73). Moderate study heterogeneity was found in the experimental treatment + CCNU/TMZ versus CCNU (*I* ^2=^ 35.59%; *p* = 0.6171) and experimental treatment versus bevacizumab (*I* ^2=^ 31.77%; *p* = 0.3759) subgroups, while considerable heterogeneity was observed in the experimental treatment versus CCNU (*I* ^2^ = 75.85%, *p* = 0.7901) subgroup.

### 3.4 Progression‐free survival

**FIGURE 3:**
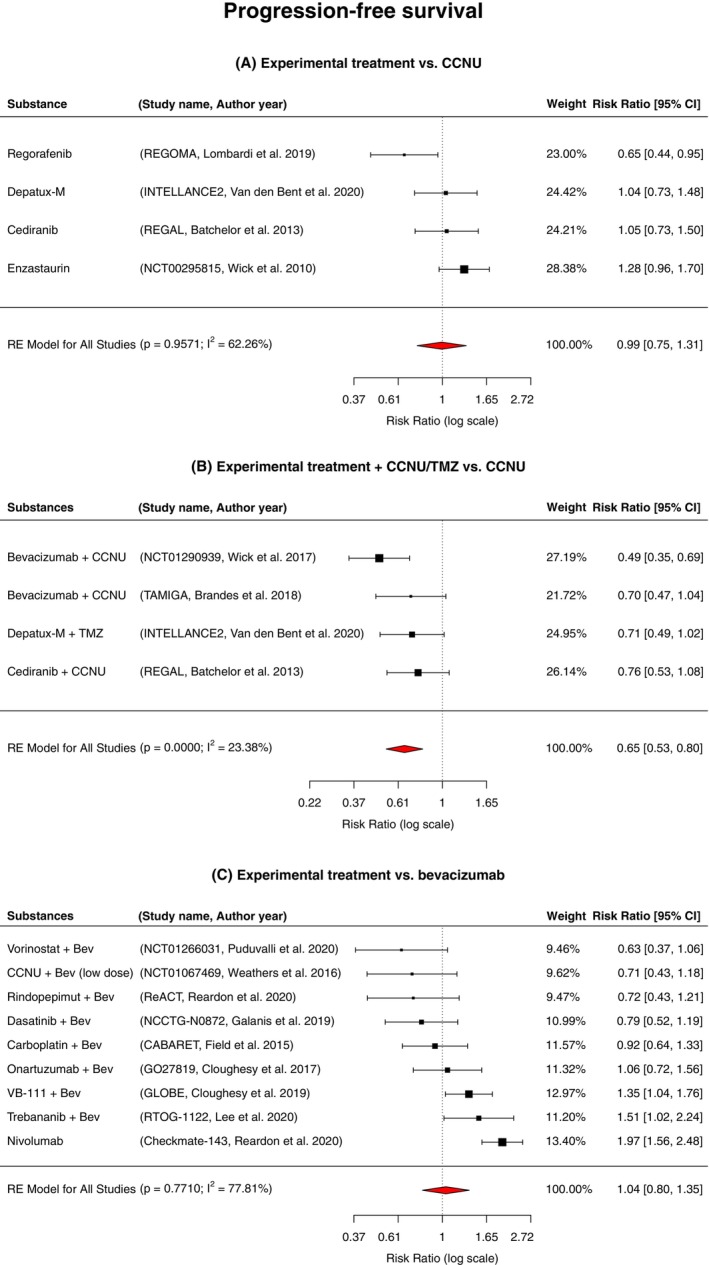
Forest plot of the pooled estimated risk ratio (red diamond) for progression‐free survival across 15 RCTs of treatment of progressive GBs with experimental treatment versus (A) CCNU monotherapy, (B) experimental treatment + CCNU/TMZ versus CCNU monotherapy and (C) experimental treatment versus bevacizumab; Bev, Bevacizumab; CCNU, Lomustine; RE, risk estimate; TMZ, Temozolomide; VB‐111, Ofranergene obadenovec.

Fifteen studies were included for the analysis of PFS. As mentioned above, these studies had analyzed 3025 patients in total. The subgroups evaluated were the same as in the analyses on OS. Of the initial 16 studies in our meta‐analysis, one trial by Brandes et al.^27^ did not provide HRs on PFS. After conducting the random‐effects meta‐analysis a 35% significant reduction in the risk of disease progression was demonstrated in the subgroup, in which patients received an experimental treatment + CCNU versus CCNU cohort (RR = 0.65, 95% CI 0.53–0.80; *p* < 0.00001, *n* = 928; Figure 3B). In this cohort, the studies included in the analysis showed only minor heterogeneity (*I* ^2^ = 23.38%; *p* = 0.2908). In the cohorts experimental treatment versus CCNU and experimental treatment versus bevacizumab, no reduction in the risk of disease progression was observed in the experimental arms (RR = 0.99, 95% CI 0.75–1.31; *p* = 0.9571, *n* = 753; Figure 3A and RR = 1.04, 95% CI 0.80–1.35; *p* = 0.7710, *n* = 1337; Figure 3C, respectively). In these two cohorts, significant moderate heterogeneity was demonstrated among the included studies (*I* ^2^ = 62.26%; *p* = 0.0479 and *I* ^2^ = 77.81%; *p* < 0.00001, respectively).

In order to assess the PFS of the separate molecular targets in the three different subgroups, we carried out stratified analyses (Figures [Link], [Link], [Link]). Only in the patient cohort that received an experimental treatment + CCNU versus the CCNU cohort (RR = 0.65, 95% CI 0.53–0.80; *p* < 0.00001, *n* = 928; Figure S5) was the risk of disease progression significantly reduced. The only study that showed a significant benefit in this cohort by prolonging PFS after receiving the combination of bevacizumab and CCNU, was the NCT01290939 trial^23^ (RR = 0.49, 95% CI 0.35–0.69; *p* < 0.00001, *n* = 437). The other studies in this cohort showed a tendency towards improved PFS, but this tendency did not reach significance. In the experimental treatment versus CCNU subgroup (Figure S4), multikinase‐inhibition with regorafenib in the REGOMA‐trial^25^ (RR 0.65, 95% CI 0.44–0.95; *p* = 0.022; *n* = 119) was significantly associated with improved PFS, while protein kinase C inhibition with enzastaurin showed an increased risk for disease progression (RR 1.28, 95% CI 0.96–1.70; *p* = 0.08; *n* = 266). In the experimental treatment versus bevacizumab subgroup, all studies failed to show a significant improvement of PFS, although HDAC‐inhibition with vorinostat and bevacizumab^37^ showed a trend towards prolonged PFS (RR 0.63, 95% CI 0.37–1.06; *p* = 0.08; *n* = 85). Experimental treatment with Ang1/2‐inhibition with trebananib combined with bevacizumab^35^ and PD‐1 inhibition with nivolumab^36^ were significantly associated with a risk of disease progression when compared to bevacizumab only (RR 1.51, 95% CI 1.05–2.24; *p* = 0.04, *n* = 115 and RR 1.97, 95% CI 1.56–2.48; *p* < 0.0001, *n* = 367, respectively).

### 3.5 Further subgroup analysis

Further stratification of all three subgroups was performed based on the following characteristics: methylated/unmethylated O^6^‐methylguanine‐DNA‐methyltransferase (MGMT) status, sex, use of steroids, ethnicity, Karnofsky Performance Status (KPS) and first relapse (Figure [Link], [Link], [Link], [Link], [Link], [Link], [Link], [Link]). Of note, not all factors were evaluable in each subgroup.

When analyzing OS, the subgroup analyses failed to demonstrate a significant benefit in any subgroup, besides in the experimental treatment + CCNU versus CCNU cohort, where a methylated MGMT promoter status was associated with improved OS (RR 0.70; 95% CI 0.50–0.99; *p* = 0.045), although this tendency did not reach significance in any of the included studies^23^, ^28^ when evaluated separately. These two studies were very homogeneous (*I* ^2^ = 0.00%).

Other factors, such as KPS, first relapse, sex, ethnicity, and steroid use did not show any statistical difference. However, there was a tendency in patients with a KPS ≤80 to have an increased risk of death in the experimental treatment versus bevacizumab subgroup, although this trend was not statistically significant.

For PFS, we again found that in the experimental treatment + CCNU versus CCNU cohort, the subgroups, that had a methylated MGMT promoter, showed a significantly prolonged PFS (RR 0.57, 95% CI 0.34–0.93; *p* = 0.0254; *I* ^2^ = 56.15%). All other factors that were examined in the subgroups did not reach significance (Figure S14).

**FIGURE 4:**
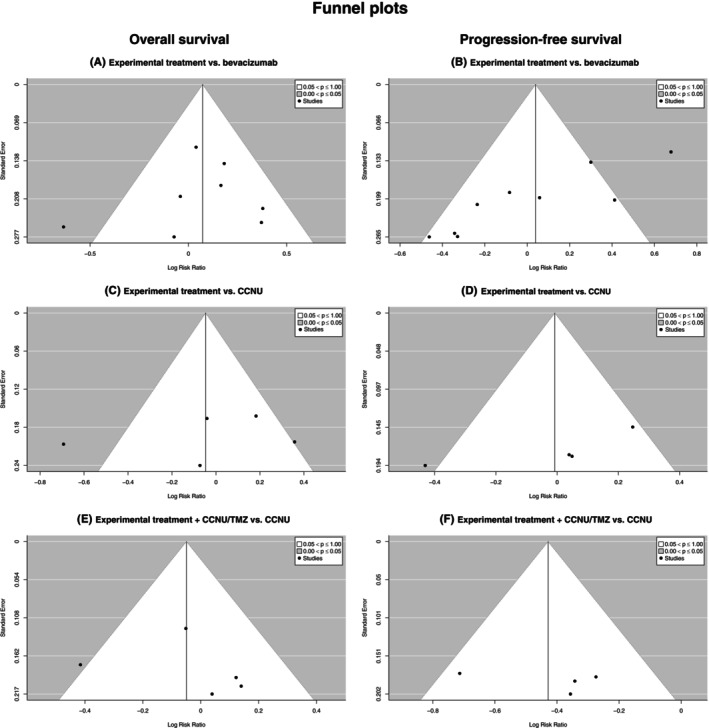
Funnel plots for risk of overall survival and disease progression depicting log risk ratio for (A), (C) and (E) OS = overall survival; (B), (D) and (F) PFS = progression‐free survival for the three different treatment cohorts each displaying an almost symmetrical distribution, indicating no publication bias.

The corresponding funnel plots depicting the risk of OS and PFS (Figure 4) show an almost symmetrical distribution. This indicates that there is no publication bias, which was also supported by the results of the Begg's (for OS: *p* = 0.4833 [experimental treatment + CCNU vs. CCNU]; *p* = 0.4833 [experimental treatment vs. CCNU]; *p* = 0.7275 [experimental treatment vs. bevacizumab]; for PFS: *p* = 1.00 [experimental treatment + CCNU vs. CCNU]; *p* = 0.3333 [experimental treatment vs. CCNU]; *p* = 0.5484 [experimental treatment vs. bevacizumab]) and Egger's (for OS: *p* = 0.5839 [experimental treatment + CCNU vs. CCNU]; *p* = 0.6301 [experimental treatment vs. CCNU]; *p* = 0.9607 [experimental treatment vs. bevacizumab]; for PFS: *p* = 0.4551 [experimental treatment + CCNU vs. CCNU]; *p* = 0.0297 [experimental treatment vs. CCNU]; *p* = 0.1025 [experimental treatment vs. bevacizumab]) tests (see Table 2).

**TABLE 2:** Results of the Begg's and Egger's test, indicating no publication bias.

	Begg's test		Egger's test	
	Overall survival	Progression‐free survival	Overall survival	Progression‐free survival
Experimental treatment + CCNU versus CCNU	*p* = 0.4833	*p* = 1.00	*p* = 0.5839	*p* = 0.4551
Experimental treatment versus CCNU	*p* = 0.4833	*p* = 0.3333	*p* = 0.6301	*p* = 0.0297
Experimental treatment versus bevacizumab	*p* = 0.7275	*p* = 0.5484	*p* = 0.9607	*p* = 0.1025

Abbreviation: CCNU, Lomustine.

## 4 DISCUSSION

Despite advances in improving our understanding of gliomagenesis and numerous treatment approaches that have been evaluated in clinical trials, the prognosis of GB patients remains poor and almost all GBs reoccur after first‐line standard of care therapy. The identification of targetable molecular alterations has led to a large number of clinical trials evaluating targeted agents with or without the combination of other agents, radiotherapy or surgery in patients with progressive GB.

This infield meta‐analysis of patients with progressive GB, treated with targeted agents is, as far as we know, the first and most extensive analysis comparing different therapeutic targets and their effects on OS and PFS.

In this meta‐analysis, we identified 16 RCTs, in which patients have received treatment with a targeted therapy either alone or in combination with another medication (CCNU, bevacizumab or temozolomide), as part of the experimental arm, compared to CCNU or bevacizumab as part of the control arm. Three studies^24^, ^27^, ^28^ analyzed trials with two experimental treatment groups.

Most studies compared bevacizumab alone to a combination with bevacizumab plus a targeted agent. Despite being the control arm in many studies on progressive GB, the use of bevacizumab in progressive GB is highly discussed considering for example the effects of bevacizumab on the blood–brain barrier shown in neuroimaging.^38^, ^39^

In two studies, bevacizumab + CCNU was further compared to CCNU alone, making the VEGF/VEGFR pathway the most frequently analyzed pathway in these trials. Of this particular subgroup, only the ReACT trial^31^ evaluating rindopepimut, the tumor‐specific EGFRvIII driver mutation vaccine, in combination with bevacizumab versus bevacizumab alone yielded a substantial benefit on OS, although it failed to show a significantly improved PFS, but a tendency was observed. This could have been due to the small sample size (73 patients in total) and potential heterogeneity of bevacizumab response assessed in the study, but can also be attributed to a potential underrepresentation of EGFRvIII in the study population, as archival tumor at initial diagnosis was used for EGFRvIII‐detection in 79% of patients.^31^, ^40^ On the basis of this trial, a more advanced trial design of personalized immunotherapies has been proposed integrating biological and clinical endpoints more carefully, including extensive tissue analysis from a recent biopsy or resection in the first place, followed by careful selection of the designated target. Furthermore, development of a treatment should ideally begin prior to resection, followed by intensive minimal‐invasive monitoring with liquid biopsies, then, after the resection multivariable analysis of the resected tissue with either continuation, modification or end of treatment as a consequence should follow.^40^ As a next step, an optimized trial design needs to be taken into account upfront carefully, as precision medicine requires defining clinical trial populations on an even more granular level compared to other RCTs. This results in stratifying patient populations into smaller, treatment‐eligible subgroups to better address the increasing complexity of molecularly directed therapies. In order to address this challenge adequately, more innovative trial designs such as basket or umbrella trials, the integration of real‐world data and the development of improved predictive preclinical models will be a crucial aspect.^41^ Moreover, ethical concerns may arise in RCTs in personalized medicine when there is a valid assumption that a patient could benefit more from a specific therapy due to its molecular mechanism of action. In such cases, administering a potentially inferior treatment to act as a control could violate ethical standards and undermine patient consent to participate in the trial, necessitating careful consideration of ethical implications when designing and conducting RCTs in personalized medicine.^42^

However, despite a careful trial design, in the ACT‐IV phase 3 trial, in which patients, who had been newly diagnosed with an EGFRvIII‐expressing GB, received treatment with rindopepimut and temozolomide, rindopepimut failed to increase survival in this patient cohort,^43^ which questions results from the previous ReAct trial in progressive GB.

All other experimental treatments compared to bevacizumab assessed in this meta‐analysis failed to show a significant tendency towards improved OS.^29^, ^32^, ^33^, ^34^, ^35^, ^36^, ^37^ In regards to PFS for this particular subgroup, VB‐111 (ofranergene obadenovec)^34^ + bevacizumab, trebananib^35^ + bevacizumab and nivolumab^36^ even resulted in a risk for diminished PFS compared to their respective control with bevacizumab only. While it has been discussed for trebananib that the addition to bevacizumab seemed to be detrimental,^35^ it has been claimed for VB‐111 that the different treatment regimen in the phase III study (with a lack of VB‐111 monotherapy priming compared to the previous phase II study,^44^ which had yielded more promising results), might have accounted for the unfavorable outcome in this trial.^34^

Of note, the Checkmate‐143 trial (nivolumab vs. bevacizumab)^36^ did not meet its primary endpoint of superior survival with checkpoint blockade. The results of this study have been extensively discussed and mostly been attributed to the low levels of PD‐L1 expression in this trial, as this has been shown to be a predictive biomarker of treatment response to immune checkpoint blockade across various other cancer types.^45^, ^46^ Furthermore, a high mutational load in a tumor has also been associated with an increase of tumor‐specific neoantigens which are able to result in a solid anti‐tumor response, which has led the focus of a recent phase I trial to investigate immune checkpoint inhibition in hypermutated progressive glioma (NCT02658279). Also, it has been proposed that the amount of CD4+ and CD8+ T‐cells might be too low in the GB tumor microenvironment to result in robust treatment responses to immune checkpoint blockade.^45^ As a result, more and more clinical trials are investigating immune checkpoint inhibition in combination with other targeted therapies and/or vaccines in order to increase T‐cell recruitment into the tumor microenvironment, which are already published^47^, ^48^, ^49^ or still under investigation (e.g., NCT03893903, NCT04116658).

When focusing on trials with CCNU as a control versus targeted agents in combination with either TMZ or CCNU, the only trial that led to an improvement in OS was the INTELLANCE 2/EORTC 1410 study. This study investigated treatment with Depatux‐M alone and with temozolomide compared to temozolomide or lomustine in progressive GB with EGFR amplification. The combination of Depatux‐M with TMZ yielded a significant improvement of OS compared to the control arm in the long‐term follow‐up, irrespective of the MGMT‐promoter methylation status.^28^ However, Depatux‐M monotherapy showed no evidence of efficacy. Importantly, the recently published companion phase III trial INTELLANCE 2 evaluating Depatux‐M in combination with standard chemo‐irradiation with TMZ in newly diagnosed EGFR‐amplified GB patients was discontinued for futility after an interim analysis that did not demonstrate any benefit in regards to OS.^50^ Therefore, the authors concluded that the results of INTELLANCE 2 may be questioned by the phase III trial, but that a more favorable subgroup of patients with progressive GB might still benefit from this particular combination therapy,^28^ which could not be proven to date.

Not surprisingly, the combination of CCN + bevacizumab versus CCNU alone resulted in a benefit regarding PFS in the NCT01290939 trial,^23^ but results on bevacizumab in GB have been questioned besides proven effects on radiation necrosis.^38^, ^39^ Interestingly, the TAMIGA study evaluating the same combination did not reach significance for improved PFS, but it has to be noted that the trial setup was different, treating patients with newly diagnosed GB with a combination of chemoradiation with TMZ and bevacizumab and then randomizing patients at recurrence to the combination therapy stated.^22^ Furthermore, the trial had been prematurely terminated due to a high drop‐out rate during first‐line treatment of recruited patients, putting the study at risk for potentially underpowered inferential statistical analyses.^22^ All other combinations of targeted agents + CCNU versus CCNU showed a tendency towards improved PFS which did not reach significance.

When comparing targeted agent versus CCNU, the REGOMA trial investigating the oral multikinase inhibitor regorafenib resulted in improved OS and PFS rates for patients treated in the experimental arm of this study.^25^ However, this trial has been criticized as it only included a small number of patients, and the prognostic factors such as age, MGMT‐promoter methylation status, steroid dependency and time until first relapse favored the experimental treatment arm, which led to comparably short survival rates in the CCNU control arm. Furthermore, it has to be mentioned that the GB adaptive, global, innovative learning environment (GBM AGILE, an international, seamless Phase II/III response adaptive randomization platform trial designed to evaluate multiple therapies in newly diagnosed and progressive GB) recently announced that enrollment for the regorafenib arm was stopped. This statement came after an interim analysis had been conducted, which had demonstrated that there was a low probability of sufficient improvement in OS as compared with randomized controls.^51^

In this meta‐analysis, we identified, extracted and analyzed data from many clinical trials using various targeted agents with different mechanisms of action. Only completed randomized controlled clinical trials with the highest available evidence level (phase II or phase III) were selected. Additionally, this ensured a high standard of study design, data processing, statistics, and data reporting. Moreover, most of the analyzed studies were registered trials. Due to this fact, the involved study groups, as well as industry and competent authorities provided another level of quality assurance.^16^

However, due to this strict selection of phase II and III RCTs, this meta‐analysis has inherent limitations. We decided to include only full‐text articles of these trials, which had to provide data on OS and PFS. For this reason, studies on targeted substances, that were published more recently, were not included. Moreover, the clinical heterogeneity between trials, which is shown, for example, in the greatly varying numbers of study participants (ranging from 69 to 367), needs to be taken into account when interpreting the results of this meta‐analysis. Only little data was available on different subgroups examined, such as molecular markers, sex, ethnicity, steroid use, KPS and first relapse due to the lack of reported hazard ratios, posing an additional challenge regarding a more specified subgroup analysis. Most data for subgroup analyses were based on bevacizumab trials only. It should furthermore be noted that conferring the patients' documented WHO tumor grade of the classification systems of 2006 and 2017 into the recently published WHO Classification of Tumors of the Central Nervous System of 2021 was not possible.^52^ This is of particular clinical relevance as some of the previously classified “glioblastomas” included in this analysis would now probably have to be reclassified as diffuse astrocytomas CNS‐WHO‐Grades 2, 3 or 4. As this selection of GBs according to the most recent classification was not possible, this could have led to either an under or overestimation of reported effects.

Taking everything into account, while some studies demonstrate a benefit either for OS and/or PFS, it is important to critically evaluate these results in regards to the sample size and trial design, as well as the initial distribution of prognostic factors and known underlying molecular mechanisms. According to published guidelines targeted agents are to be applied primarily in clinical trials; the results of this meta‐analysis support this approach. Furthermore, this analysis emphasizes the necessity for randomized clinical trials with a more specific and personalized design. This could involve the use of ideally recently obtained tissue to ensure that molecularly targeted structures are present in the tumor tissue, or monitoring of the treatment response closely with the emergence of liquid biopsies and as a consequence allowing a potential modification of the treatment if deemed necessary.

## AUTHOR CONTRIBUTIONS

**Franziska Maria Ippen:** Conceptualization (lead); data curation (lead); formal analysis (equal); investigation (lead); methodology (lead); project administration (lead); resources (equal); validation (lead); visualization (lead); writing – original draft (lead). **Angelika Scherm:** Data curation (equal); formal analysis (lead); investigation (equal); methodology (equal); software (equal); visualization (equal); writing – review and editing (equal). **Tobias Kessler:** Project administration (equal); validation (equal); writing – review and editing (equal). **Peter Hau:** Methodology (equal); validation (equal); writing – review and editing (equal). **Sarina Agkatsev:** Writing – review and editing (equal). **Hansjörg Baurecht:** Formal analysis (equal); methodology (equal); software (equal); validation (equal); writing – review and editing (equal). **Wolfgang Wick:** Project administration (equal); validation (equal); writing – review and editing (equal). **Helge Knüttel:** Data curation (equal); writing – review and editing (equal). **Michael F. Leitzmann:** Formal analysis (equal); methodology (equal); software (equal); validation (equal); writing – review and editing (equal). **Corinna Seliger‐Behme:** Conceptualization (equal); investigation (equal); project administration (equal); supervision (lead); writing – review and editing (equal).

## CONFLICT OF INTEREST STATEMENT

All authors declare no potential conflicts of interest.

## ETHICS STATEMENT

All procedures performed in the primary studies meta‐analyzed here involving human participants were in accordance with the ethical standards of the institutional and/or national research committee and with the 1964 Helsinki Declaration and its later amendments or comparable ethical standards.

## Supporting information


Figure S1.

Figure S2.

Figure S3.

Figure S4.

Figure S5.

Figure S6.

Figure S7.

Figure S8.

Figure S9.

Figure S10.

Figure S11.

Figure S12.

Figure S13.

Figure S14.

Data S1.

## Data Availability

Publicly available data generated by others were used by the authors. The data analyzed in this study were obtained from MEDLINE (Ovid)/Pubmed (https://pubmed.ncbi.nlm.nih.gov/), Cochrane Library (https://www.cochranelibrary.com/), ClinicalTrials.gov (https://clinicaltrials.gov/ct2/home), WHO's International Clinical Trials Registry Platform (ICTRP, https://www.who.int/clinical‐trials‐registry‐platform) and Google Scholar (https://scholar.google.com). The data extracted from the above‐mentioned sources are available upon request from the corresponding author.
