# Supplementary material for: Targeted agents in patients with progressive glioblastoma—A systematic meta‐analysis of randomized clinical trials
Source: Cancer Med. 2024 Jun 21;13(12):e7362. doi: 10.1002/cam4.7362 (PMC11192969; doi:10.1002/cam4.7362)
Supplement: Supplementary file 1 — Figure S1. [file CAM4-13-e7362-s005.pdf]

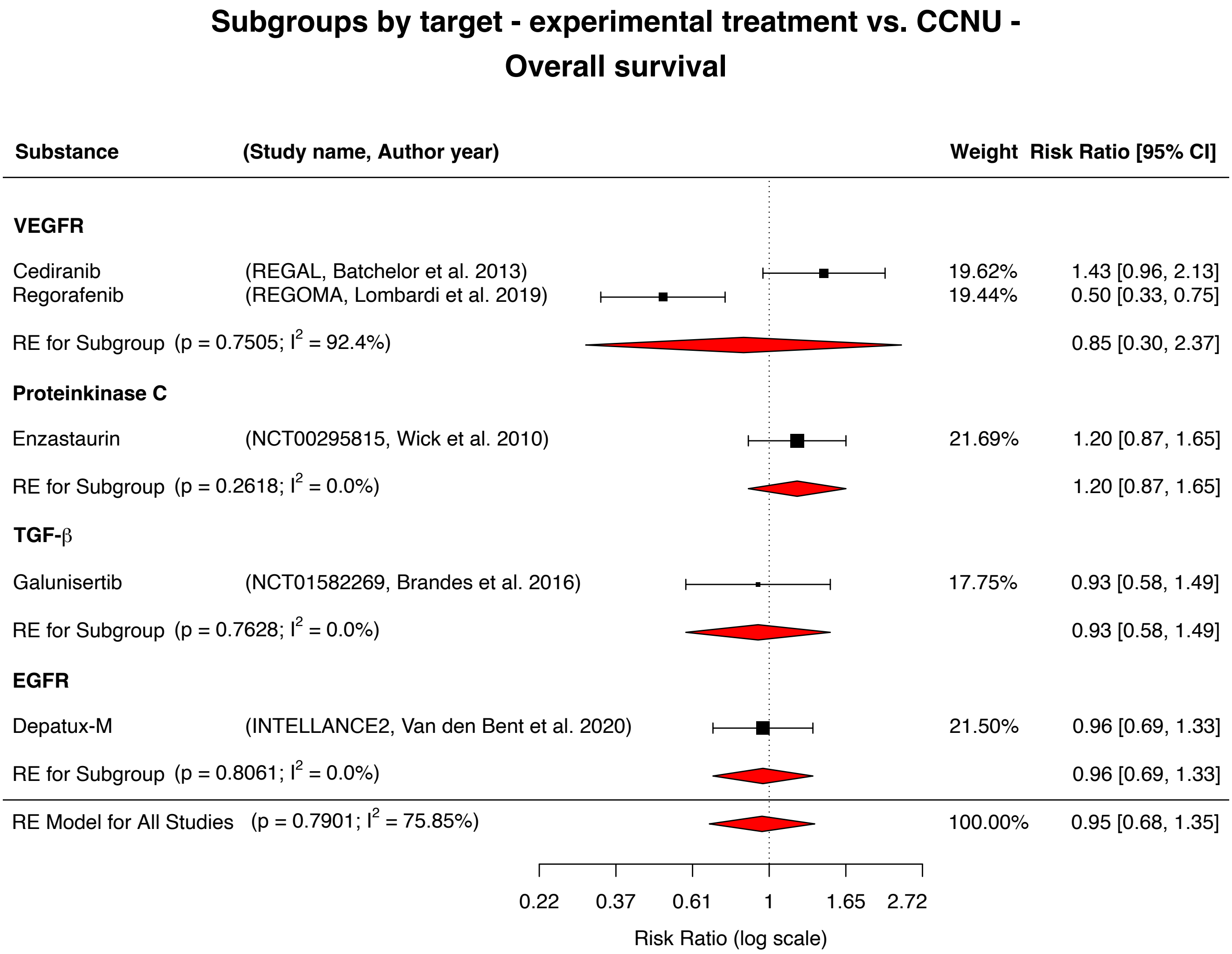

SUPPLEMENTARY FIGURE 1. Forest plot presenting the subsequent subgroup analysis by target of the pooled estimated risk ratio (red diamond) for overall survival for patients treated with experimental treatment vs. CCNU monotherapy; Abbreviations: EGFR= Epidermal growth factor receptor; CCNU= Lomustine; RE= risk estimate; TGF-β= Transforming growth factor beta; VEGF= Vascular endothelial growth factor; Treatment for the multikinase-inhibitors regorafenib and cediranib was summarized under VEGF-inhibition, although targeting multiple kinases
