# Supplementary material for: Targeted agents in patients with progressive glioblastoma—A systematic meta‐analysis of randomized clinical trials
Source: Cancer Med. 2024 Jun 21;13(12):e7362. doi: 10.1002/cam4.7362 (PMC11192969; doi:10.1002/cam4.7362)
Supplement: Supplementary file 3 — Figure S3. [file CAM4-13-e7362-s014.pdf]

Subgroups by target - experimental treatment vs. bevacizumab - Overall survival

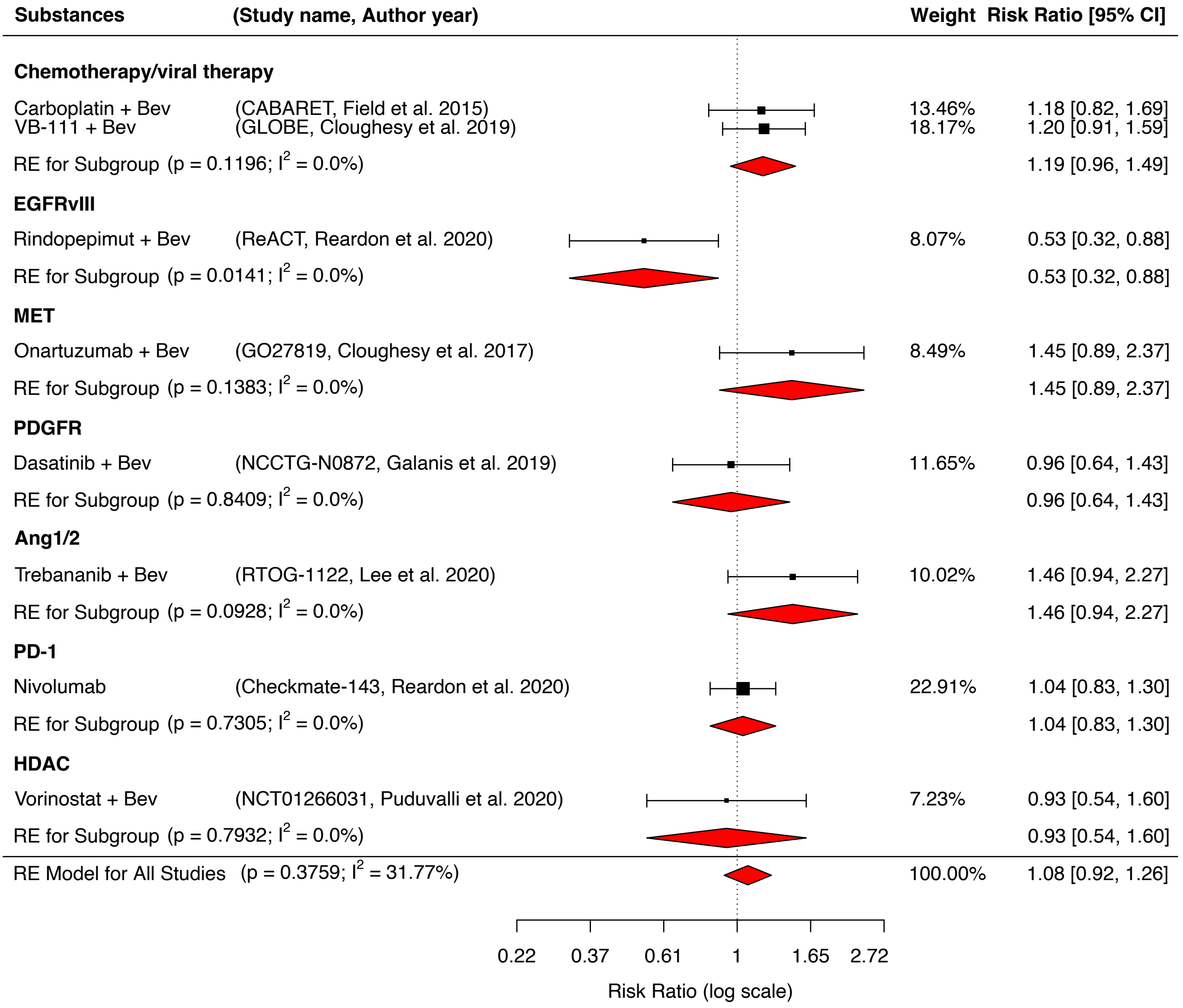

SUPPLEMENTARY FIGURE 3. Forest plot presenting the subsequent subgroup analysis by target of the pooled estimated risk ratio (red diamond) for overall survival for patients treated with experimental treatment vs. Bevacizumab; Abbreviations: Ang1/2= Angiopoietins 1 and 2; BEV= Bevacizumab; CCNU= Lomustine; EGFRvIII= Epidermal growth factor receptor variant three; HDAC= Histone-Deacetylase; MET= mesenchymal-epithelial transition receptor tyrosine kinase; PD-1= Programmed cell death protein 1; PDGFR= Platelet-derived growth factor receptor; RE= risk estimate; VB-111= Ofranergene obadenovec
