# Supplementary material for: Targeted agents in patients with progressive glioblastoma—A systematic meta‐analysis of randomized clinical trials
Source: Cancer Med. 2024 Jun 21;13(12):e7362. doi: 10.1002/cam4.7362 (PMC11192969; doi:10.1002/cam4.7362)
Supplement: Supplementary file 5 — Figure S5. [file CAM4-13-e7362-s004.pdf]

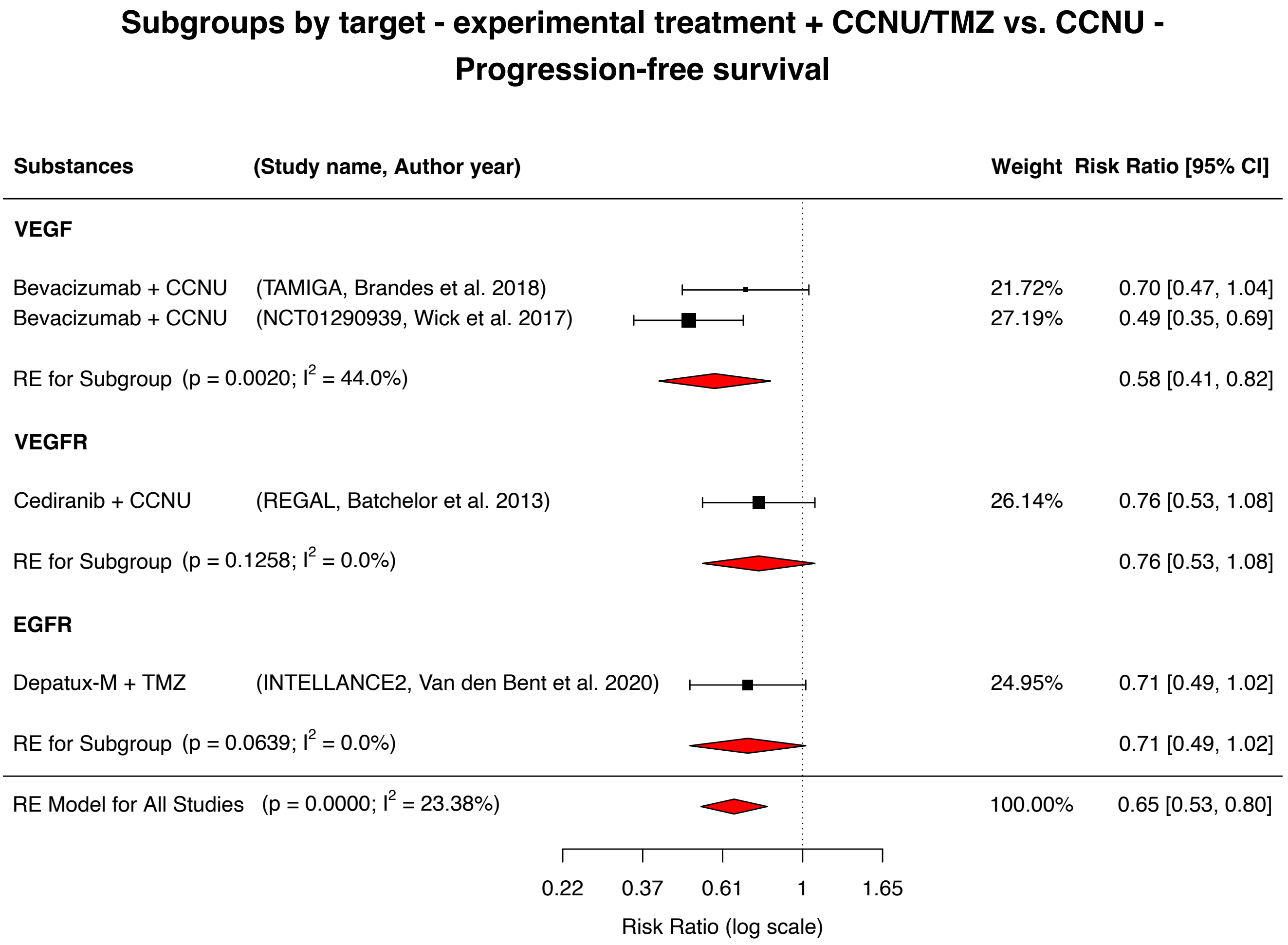

SUPPLEMENTARY FIGURE 5. Forest plot of the subgroup analysis by target of the pooled estimated risk ratio (red diamond) for progression-free for patients treated with experimental treatment + CCNU/TMZ vs. CCNU monotherapy; Abbreviations: EGFR= Epidermal growth factor receptor; CCNU= Lomustine; RE= risk estimate; TMZ= Temozolomide; VEGF= Vascular endothelial growth factor; VEGF= Vascular endothelial growth factor receptor; Treatment for the multikinase-inhibitor cediranib was summarized under VEGF-inhibition, although targeting multiple kinases
