# Supplementary material for: Targeted agents in patients with progressive glioblastoma—A systematic meta‐analysis of randomized clinical trials
Source: Cancer Med. 2024 Jun 21;13(12):e7362. doi: 10.1002/cam4.7362 (PMC11192969; doi:10.1002/cam4.7362)
Supplement: Supplementary file 6 — Figure S6. [file CAM4-13-e7362-s002.pdf]

Subgroups by target - experimental treatment vs. bevacizumab - Progression-free survival

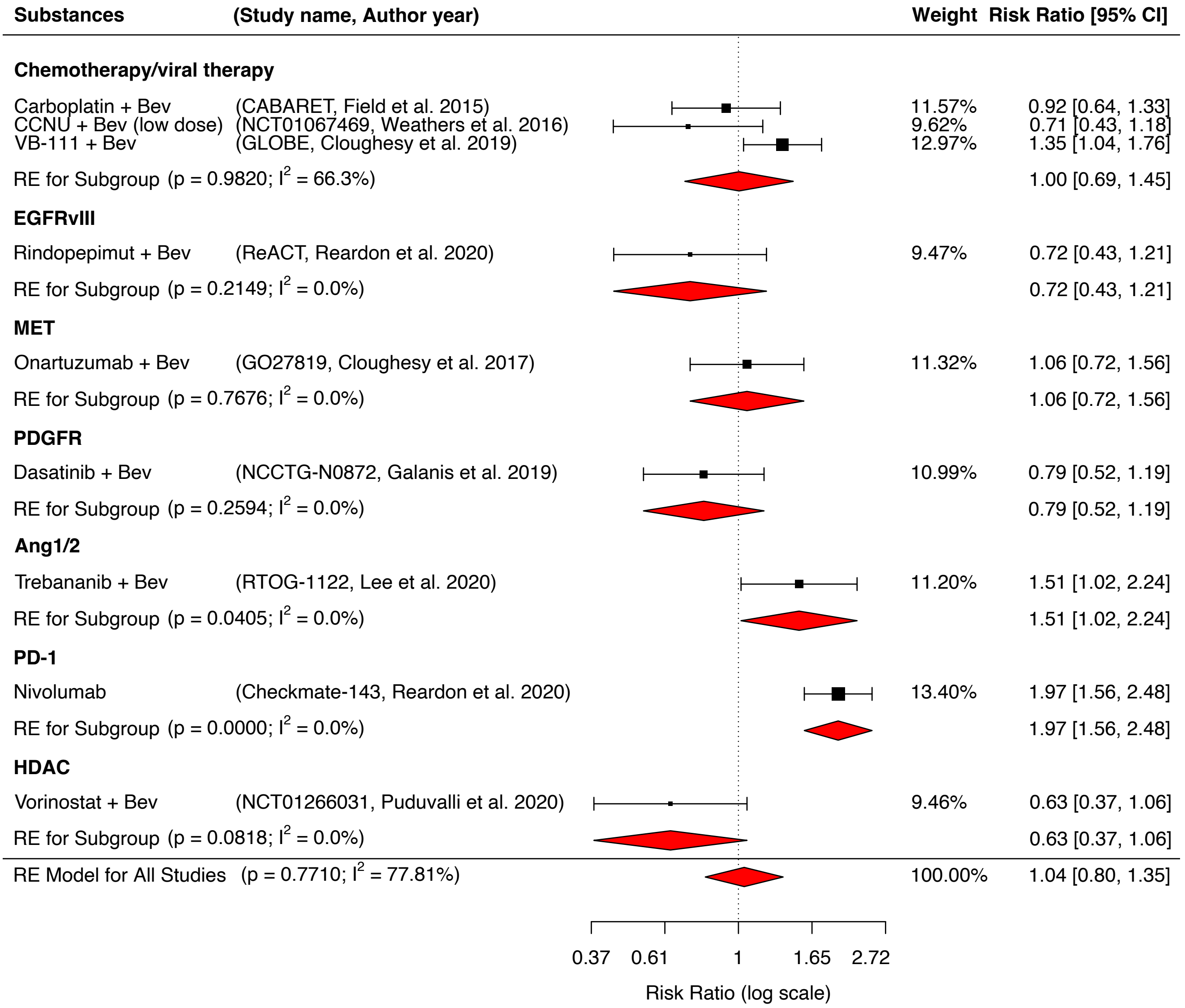

SUPPLEMENTARY FIGURE 6. Forest plot of the subgroup analysis by target of the pooled estimated risk ratio (red diamond) for progression-free for patients treated with experimental treatment vs. Bevacizumab; Abbreviations: Ang1/2= Angiopoietins 1 and 2; BEV= Bevacizumab; CCNU= Lomustine; EGFRvIII= Epidermal growth factor receptor variant three; HDAC= Histone-Deacetylase; MET= mesenchymal–epithelial transition receptor tyrosine kinase; PD-1= Programmed cell death protein 1; PDGFR= Platelet-derived growth factor receptor; RE= risk estimate; VB-111= Ofranergene obadenovec
