# Supplementary material for: Targeted agents in patients with progressive glioblastoma—A systematic meta‐analysis of randomized clinical trials
Source: Cancer Med. 2024 Jun 21;13(12):e7362. doi: 10.1002/cam4.7362 (PMC11192969; doi:10.1002/cam4.7362)
Supplement: Supplementary file 7 — Figure S7. [file CAM4-13-e7362-s013.pdf]

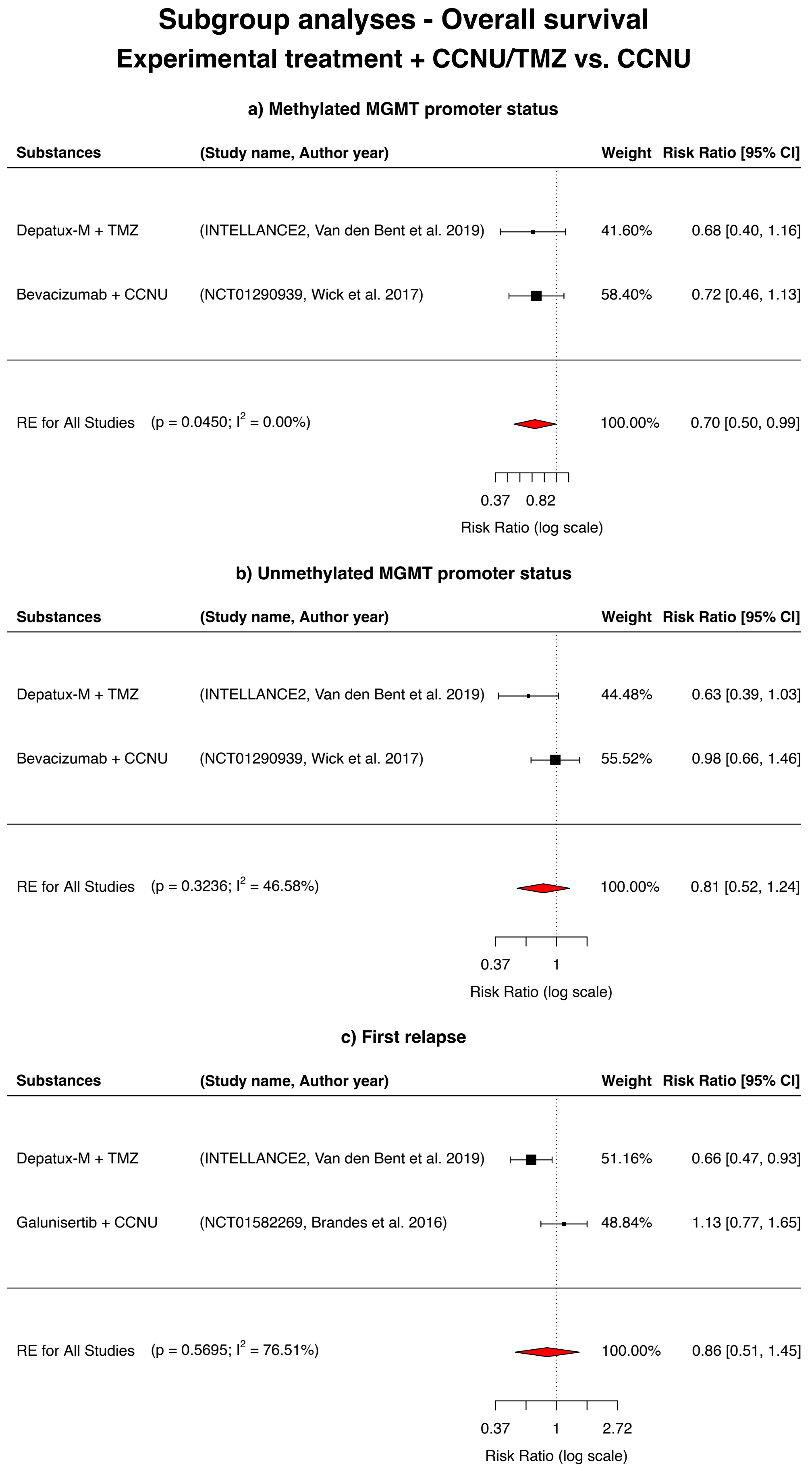

SUPPLEMENTARY FIGURE 7. Forest plots of the subsequent subgroup patient analyses of the pooled estimated risk ratio (red diamond) for overall survival for patients treated with experimental treatment + CCNU/TMZ vs. CCNU monotherapy. Abbreviations: MGMT = O^6-methylguanin-DNA-methyltransferase; CCNU= Lomustine; RE= risk estimate; TMZ= Temozolomide
