# Supplementary material for: Targeted agents in patients with progressive glioblastoma—A systematic meta‐analysis of randomized clinical trials
Source: Cancer Med. 2024 Jun 21;13(12):e7362. doi: 10.1002/cam4.7362 (PMC11192969; doi:10.1002/cam4.7362)
Supplement: Supplementary file 8 — Figure S8. [file CAM4-13-e7362-s001.pdf]

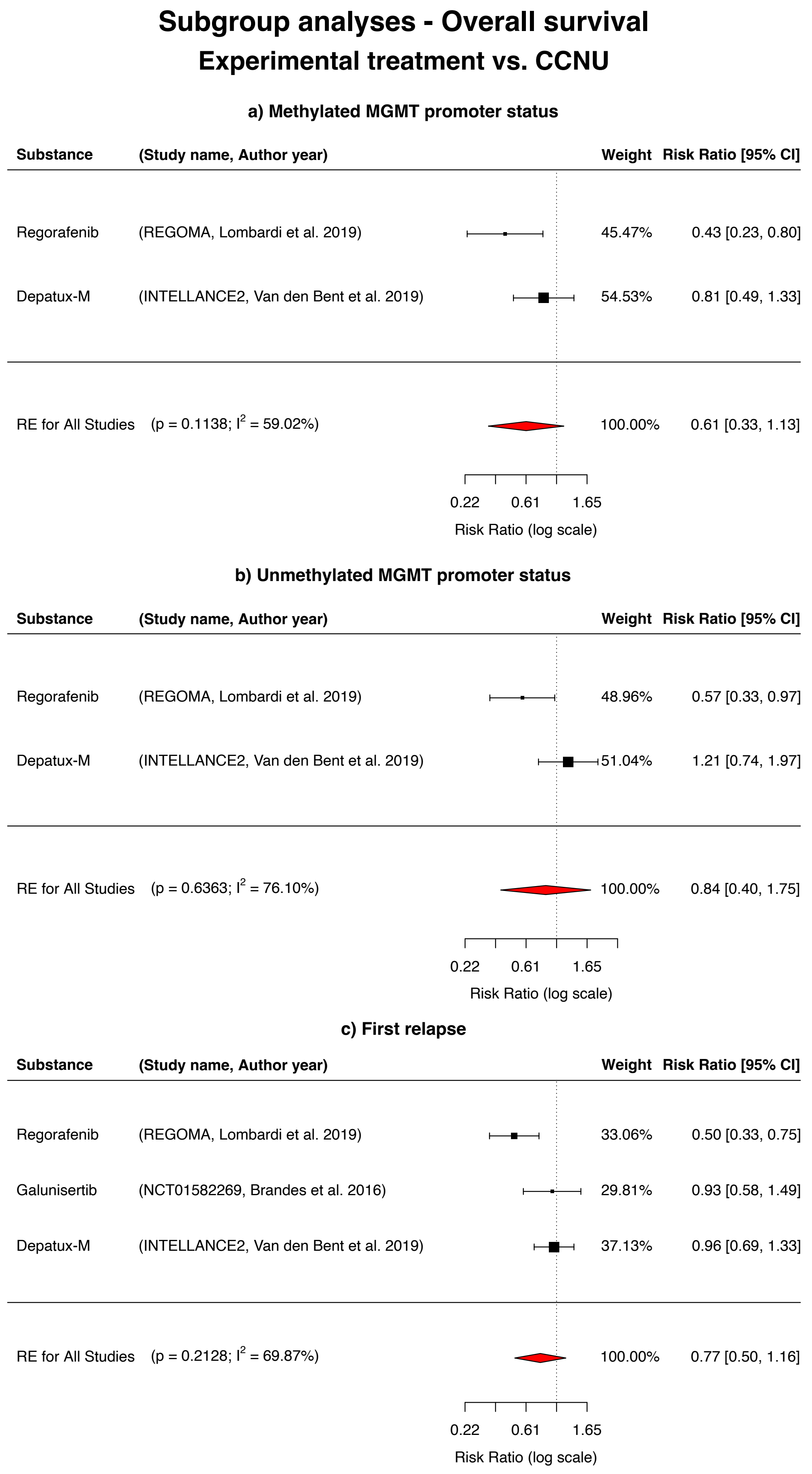


**SUPPLEMENTARY FIGURE 8.** Forest plots of the subsequent subgroup patient analyses of the pooled estimated risk ratio (red diamond) for overall survival for patients treated with experimental treatment vs. CCNU monotherapy. Abbreviations: MGMT= $O^6$-methylguanin-DNA-methyltransferase; CCNU= Lomustine; RE= risk estimate
