# Supplementary material for: Targeted agents in patients with progressive glioblastoma—A systematic meta‐analysis of randomized clinical trials
Source: Cancer Med. 2024 Jun 21;13(12):e7362. doi: 10.1002/cam4.7362 (PMC11192969; doi:10.1002/cam4.7362)
Supplement: Supplementary file 9 — Figure S9. [file CAM4-13-e7362-s006.pdf]

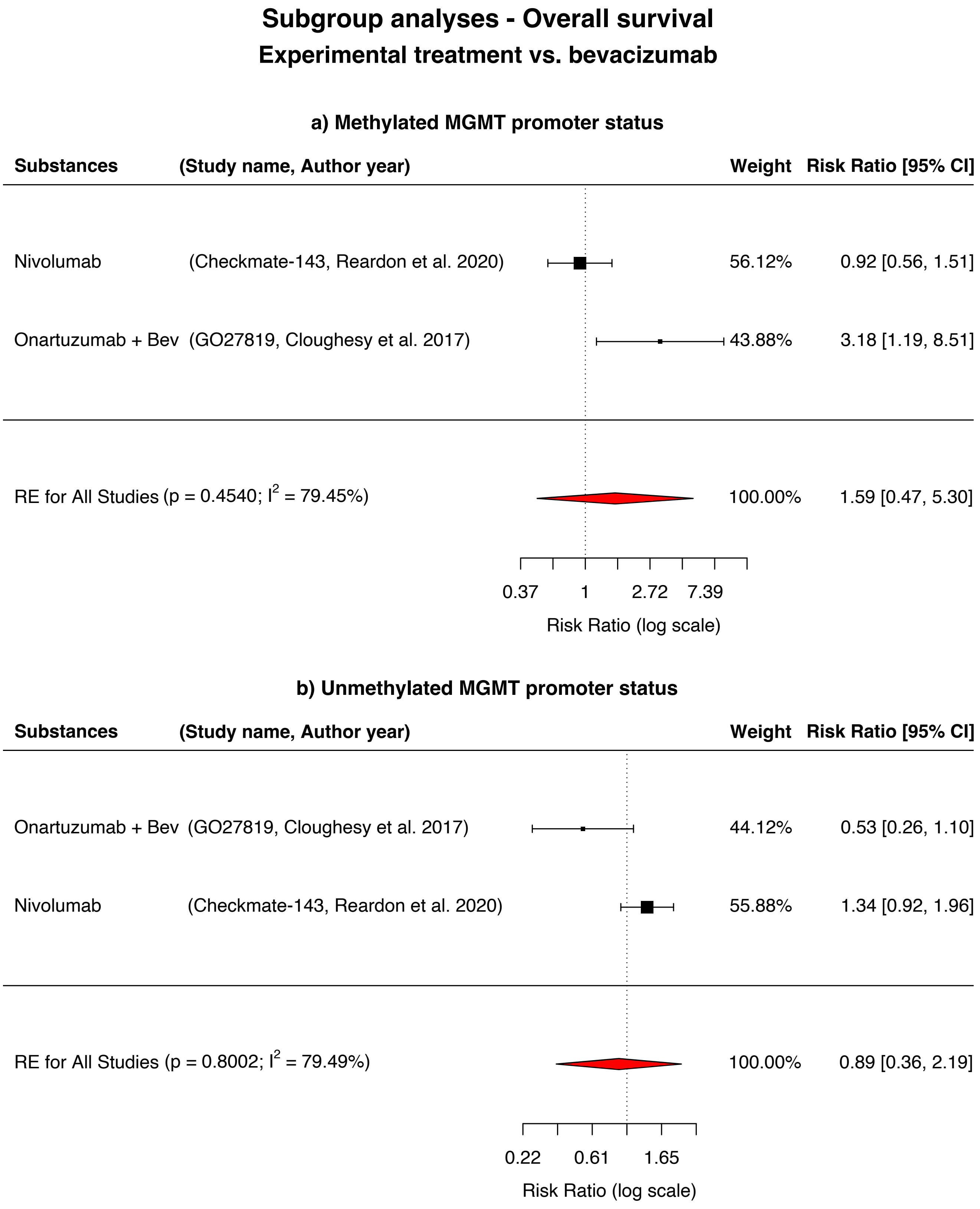

SUPPLEMENTARY FIGURE 9. Forest plots of the subsequent subgroup patient analyses (a) methylated and b) unmethylated MGMT promoter) of the pooled estimated risk ratio (red diamond) for overall survival for patients treated with experimental treatment vs. bevacizumab. Abbreviations: Bev= bevacizumab; MGMT = O^6-methylguanin-DNA-methyltransferase; RE= risk estimate
