# Supplementary material for: Targeted agents in patients with progressive glioblastoma—A systematic meta‐analysis of randomized clinical trials
Source: Cancer Med. 2024 Jun 21;13(12):e7362. doi: 10.1002/cam4.7362 (PMC11192969; doi:10.1002/cam4.7362)
Supplement: Supplementary file 10 — Figure S10. [file CAM4-13-e7362-s008.pdf]

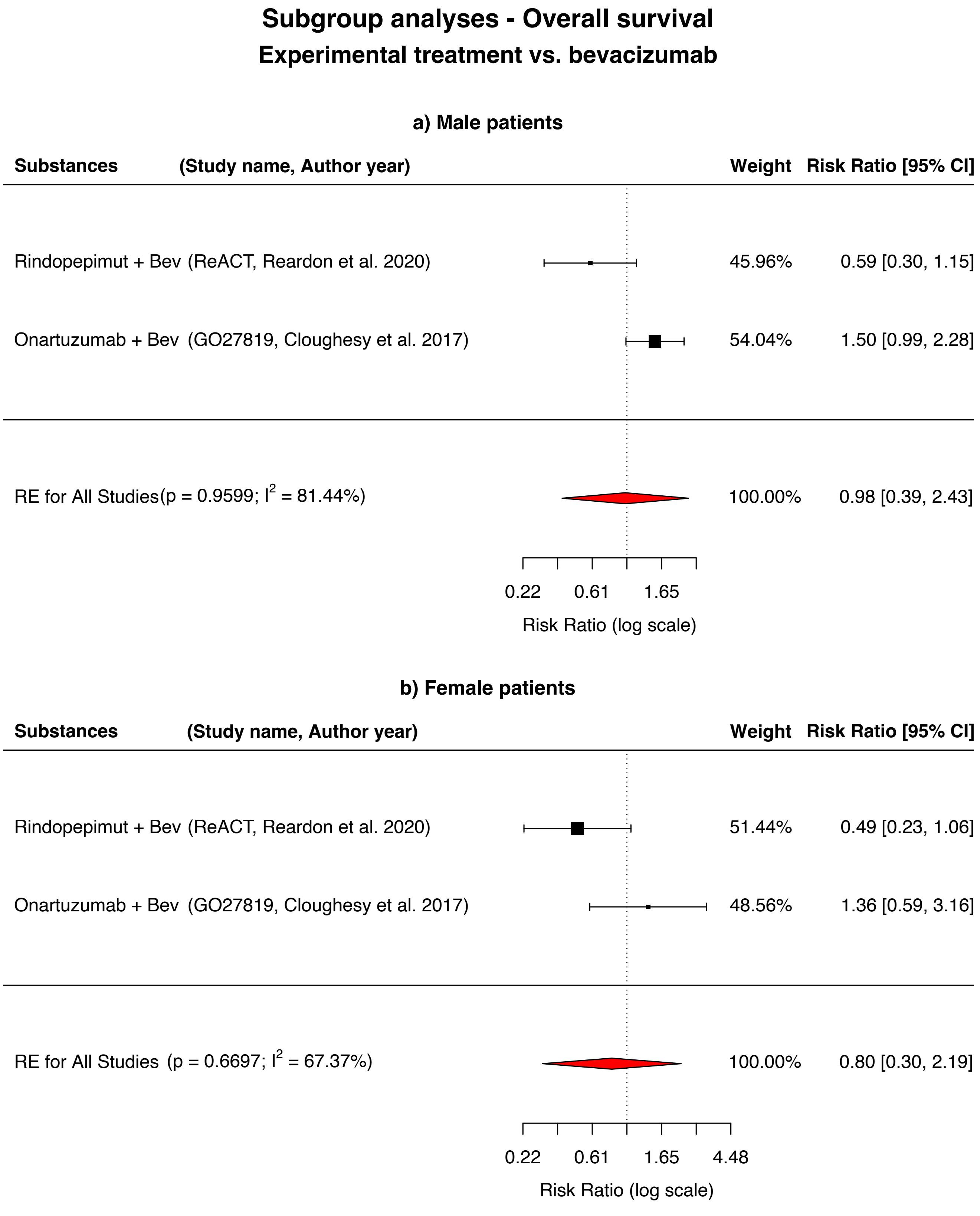

SUPPLEMENTARY FIGURE 10. Forest plots of the subsequent subgroup patient analyses (a) male and b) female patients) of the pooled estimated risk ratio (red diamond) for overall survival for patients treated with experimental treatment vs. bevacizumab. Abbreviations: Bev= bevacizumab; RE= risk estimate
