# Supplementary material for: Targeted agents in patients with progressive glioblastoma—A systematic meta‐analysis of randomized clinical trials
Source: Cancer Med. 2024 Jun 21;13(12):e7362. doi: 10.1002/cam4.7362 (PMC11192969; doi:10.1002/cam4.7362)
Supplement: Supplementary file 11 — Figure S11. [file CAM4-13-e7362-s015.pdf]

Subgroup analyses - Overall survival
Experimental treatment vs. bevacizumab

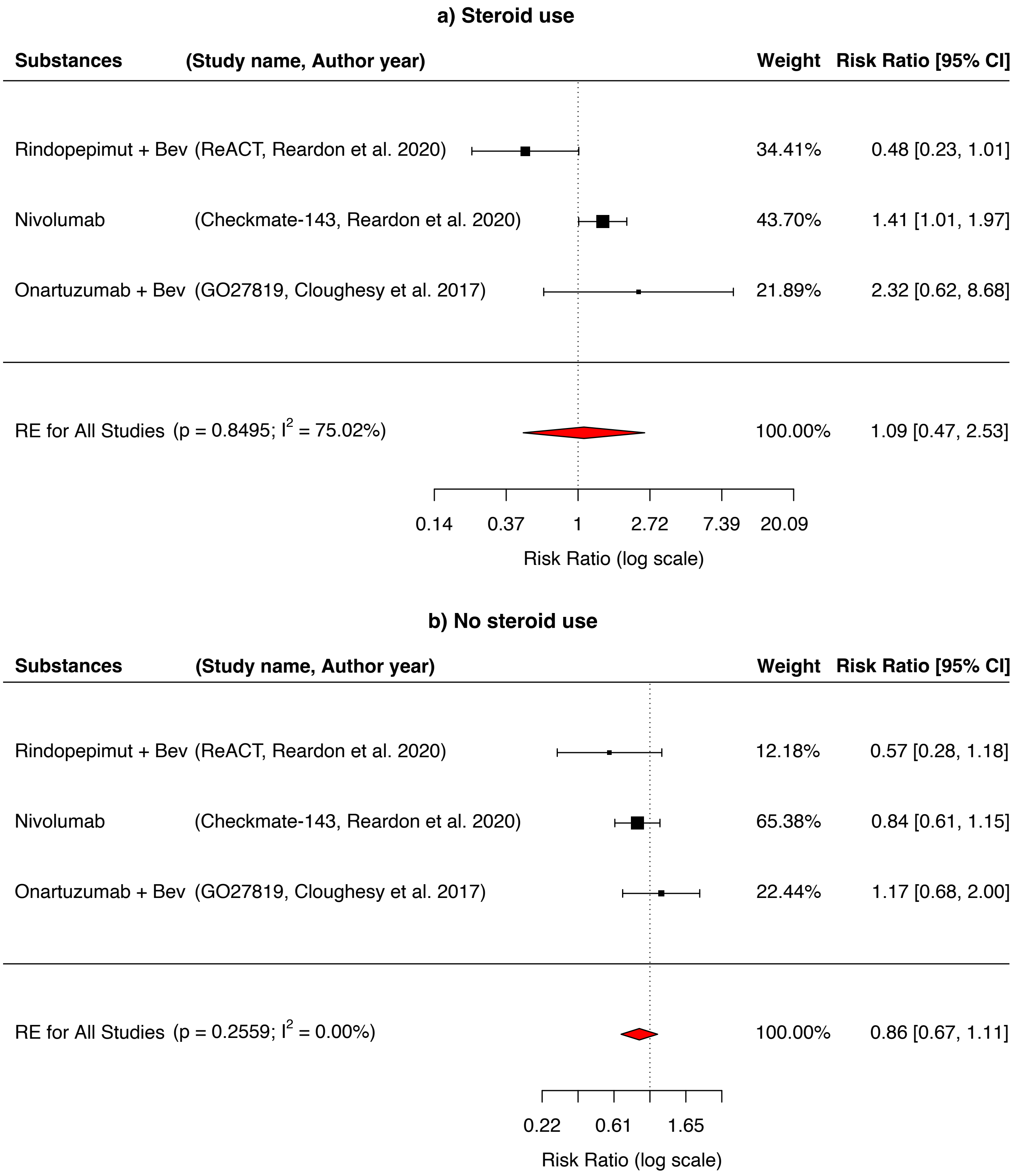

SUPPLEMENTARY FIGURE 11. Forest plots of the subsequent subgroup patient analyses (a) steroid use and b) no steroid use) of the pooled estimated risk ratio (red diamond) for overall survival for patients treated with experimental treatment vs. bevacizumab. Abbreviations: Bev= bevacizumab; RE= risk estimate
