# Supplementary material for: Targeted agents in patients with progressive glioblastoma—A systematic meta‐analysis of randomized clinical trials
Source: Cancer Med. 2024 Jun 21;13(12):e7362. doi: 10.1002/cam4.7362 (PMC11192969; doi:10.1002/cam4.7362)
Supplement: Supplementary file 13 — Figure S13. [file CAM4-13-e7362-s012.pdf]

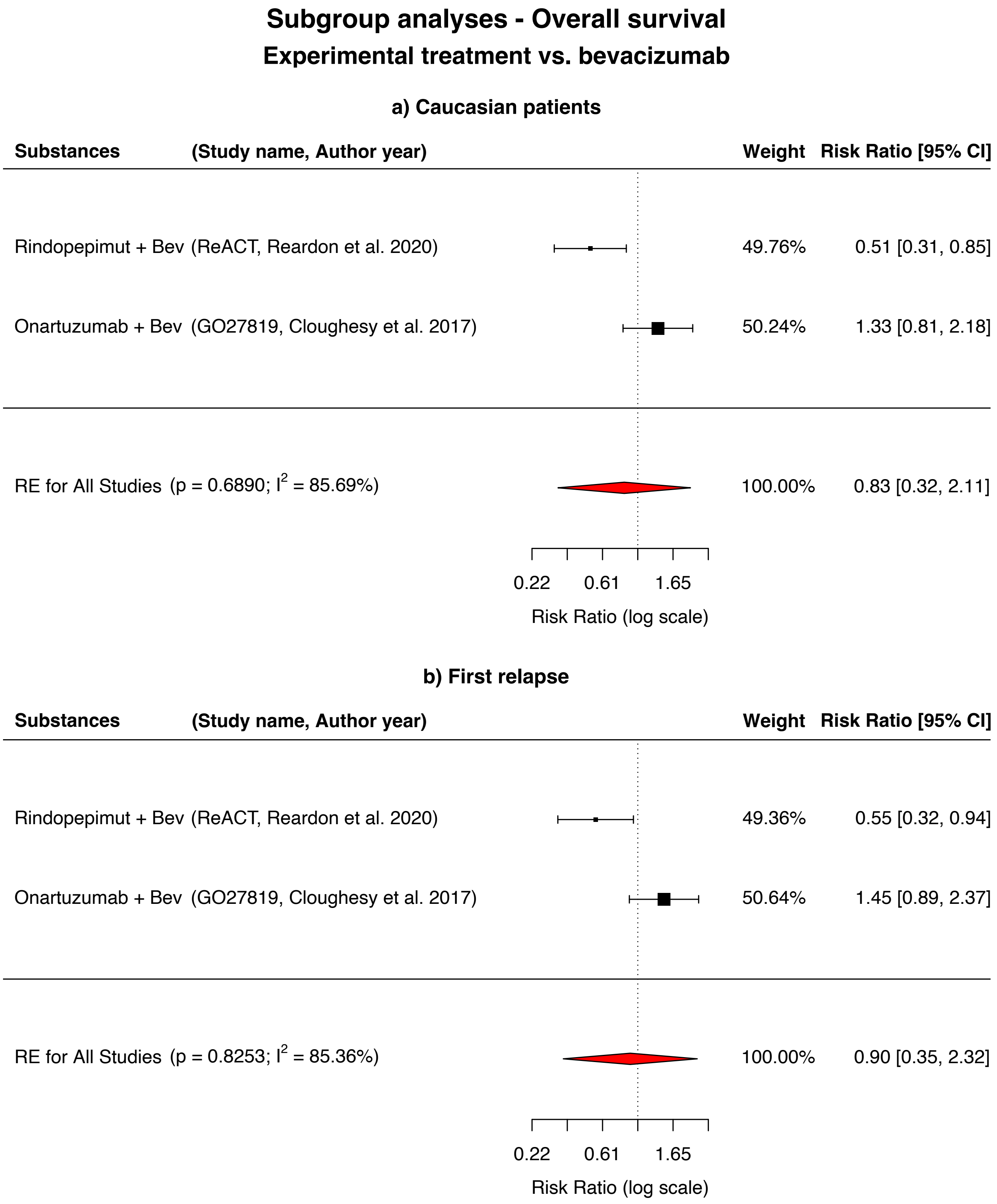


**SUPPLEMENTARY FIGURE 13.** Forest plots of the subsequent subgroup patient analyses (a) Caucasian patients and b) first relapse) of the pooled estimated risk ratio (red diamond) for overall survival for patients treated with experimental treatment vs. bevacizumab. Abbreviations: Bev= bevacizumab; RE= risk estimate
