# Supplementary material for: Targeted agents in patients with progressive glioblastoma—A systematic meta‐analysis of randomized clinical trials
Source: Cancer Med. 2024 Jun 21;13(12):e7362. doi: 10.1002/cam4.7362 (PMC11192969; doi:10.1002/cam4.7362)
Supplement: Supplementary file 14 — Figure S14. [file CAM4-13-e7362-s010.pdf]

Subgroup analyses - Progression-free survival

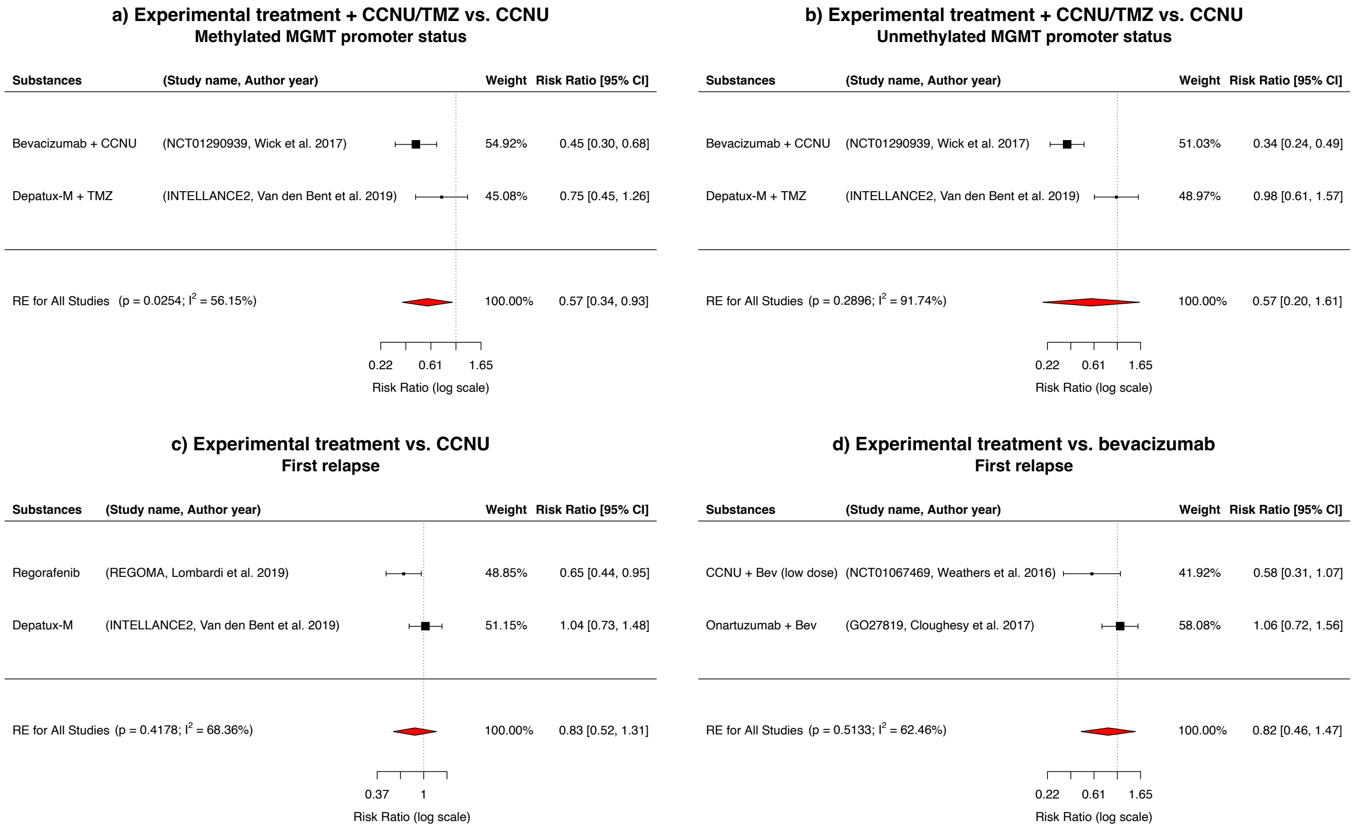

SUPPLEMENTARY FIGURE 14. Forest plots of the subsequent subgroup patient analyses of the pooled estimated risk ratio (red diamond) for progression-free survival for the designated treatment groups. Abbreviations: BEV= bevacizumab; MGMT = O^6-methylguanin-DNA-methyltransferase; CCNU= Lomustine; RE= risk estimate; TMZ= Temozolomide
